# Supplementary material for: Chromothripsis during telomere crisis is independent of NHEJ, and consistent with a replicative origin
Source: Genome Res. 2019 May;29(5):737–49. doi: 10.1101/gr.240705.118 (PMC6499312; doi:10.1101/gr.240705.118)
Supplement: Supplemental Material [file supp_gr.240705.118_Supplemental_file_1.zip › contigs/annotated_contigs/DB79/contig.2.DB79_length_683_mean_cov_6.58857979502.docx]

**DB79_length_683_mean_cov_6.58857979502**

```
AATCCCAGCACTTTGGGAGGCTGAGGCGGGCGGATCAGAGGTCAGGAGTTTGAGACAACCTGGTCAACATGGTGAAATCCCGTCTCTAC
 >chr7:116028401-116028796 + E=6e-223
TAAAAAGACAAAAATTAGCCAGGCGTGGTGGCAGGTGCCTGTAATACCAGCTACTTGGGAGGCTAAGGCAGGAGAATCTCTTGAAATCA

GAAGGTGGAGGTTGCAGTGAGCCAGGAGCACTCCACTGCACTCACTCCAGCCTGGGCAACAAGAGCAAAAGTCTGTCTCAAAAATAAAT

AAATACATAAAAATACTAAAAAAAGAAAAAATCTCAGTAGCAACACAACATTATCCTATTTAAATTTTAATATGTACTTGAACTAATGC

TTTAGCCGAATTTACTTCTATAGCTCAAAAAAATAA|AAA|GTATTGTCCACCAAATTTTTATAAATCTCCAAACACCCACAACACAAT
                                     >chr7:115668328-115668619 + E=3e-163
TCTACAGATTTCTCTAATTAGCTGGCATTCACAATTCTATATAATAAGGAATATTTGCGTAAATTAGTTTCTTGCTGTTTTTCTTTTCC

TACAAGACTACTGTTAACCTCTCTCTTCGTAGGTTATGACTTTGGGGATGATTGTCATTCCTACTGCTCTATGCCTCCCAGACTCTTCT

TAGTAAACACCCCCAGTAAAATCTGTGCAGGAACCATGCTTTGCACTATGCATGTGTTTTAT
```
